# Clinicopathological significance of Ki67 expression in colorectal cancer: A protocol of systematic review and meta-analysis

**DOI:** 10.1097/MD.0000000000020136

**Published:** 2020-05-15

**Authors:** Jing Li, Zhi-ye Liu, Hai-bo Yu, Qing Xue, Wen-jie He, Hai-tao Yu

**Affiliations:** a Department of Physiology, Jiamusi University School of Basic Medical Sciences; b Department of Chemotherapy and Radiotherapy; c Department of Cardiology; d Clinical Medicine of Class 7 in Grade 2016, Jiamusi University; e Department of Outpatient; f Department of Anorect, First Affiliated Hospital of Jiamusi University, Jiamusi, China.

**Keywords:** accuracy, colorectal cancer, Ki67 expression, sensitivity, specificity

## Abstract

**Background:**

This study will investigate the diagnostic accuracy of Ki67 expression in colorectal cancer (CC).

**Methods:**

A comprehensive search in electronic bibliographic databases (MEDLINE, EMBASE, Cochrane Library, Web of Science, Chinese Biomedical Literature Database, and China National Knowledge Infrastructure) will be performed from inception to the February 29, 2020 with no restrictions to the language and publication status. Two authors will examine the collected studies, extract essential data, and appraise study quality separately. If possible, we will estimate receiver operating characteristic (ROC), sensitivity and specificity by utilizing bivariate random effects and hierarchical summary ROC models.

**Results:**

This study will summarize present evidence to explore the diagnostic accuracy of Ki67 expression in CC.

**Conclusion:**

The findings of this study will clarify the diagnostic accuracy of Ki67 expression in CC.

**Systematic review registration:**

INPLASY202030009.

## 1 Introduction

Colorectal cancer (CC) is one of the most lethal cancers and prevalent malignant tumors globally.^[1,2,3,4]^ It has been reported that about 1.8 million new patients increased, and 881,000 patients were dead in 2018.^[5,6,7,8]^ It contributes approximately 10% new cases and mortality.^[2]^ Previous studies have reported that its 5-year survival rate is about 64%.^[9]^ However, the 5-year survival rate of metastatic CC is only 12%.^[2,9]^ Thus, it is very important to diagnose CC at early stage.

Previous studies have found that Ki67 expression may help to diagnose CC, although there are still inconsistent results.^[10,11,12,13,14,15,16,17,18]^ In addition, no systematic review has been explored to address this topic. Thus, this study will systematically assess the diagnostic accuracy of Ki67 expression in patients with CC.

## 2 Methods

### 2.1 Objective

This study aims to investigate the diagnostic accuracy of Ki67 expression in CC.

### 2.2 Study registration

This study has been registered on INPLASY (202030009). It has been organized following the guideline of Preferred Reporting Items for Systematic Reviews and Meta-Analysis Protocol statement.^[19]^

### 2.3 Eligibility criteria

#### 2.3.1 Types of studies

All case-controlled studies (CCSs) that explored the diagnostic accuracy of Ki67 expression in CC will be included. We will not apply restrictions to the basis of language of publications.

#### 2.3.2 Types of participants

We will include CCSs that compare Ki67 expression between CC participants with CC tissues and normal adjacent tissues. We will not employ any restrictions to the age, sex, race, and CC severity.

#### 2.3.3 Type of index test

Index test: We will include studies that specify the index test utilized Ki67 expression to diagnose potential patients with CC.

Reference test: Any patients with histological-proven CC will be included.

#### 2.3.4 Types of outcome measurements

Primary outcomes are sensitivity and specificity. Secondary outcomes are positive likelihood ratio, negative likelihood ratio, and diagnostic odds ratio.

### 2.4 Data sources and search strategy

#### 2.4.1 Electronic searches

A comprehensive search for associated articles in electronic bibliographic databases (MEDLINE, EMBASE, Cochrane Library, Web of Science, Chinese Biomedical Literature Database, and China National Knowledge Infrastructure) will be carried out from inception to the February 29, 2020. There will be no language and publication status restrictions. We will build search strategy sample for Cochrane Library (Table 1), and we will adapt similar search strategies for other databases.

**Table 1:**
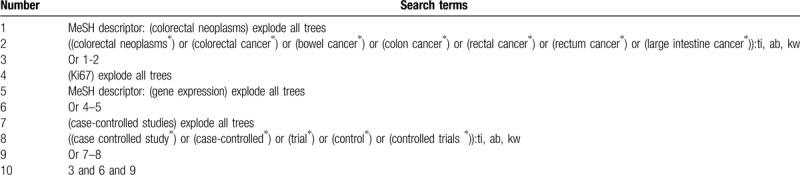
Search strategy for Cochrane Library.

#### 2.4.2 Other resources

We will check and obtain potential studies from clinical trial registry, conference abstracts, and reference lists of relevant reviews.

### 2.5 Data collection and analysis

#### 2.5.1 Selection of studies

All searched citations will be imported into EndNote 7.0 software: organization, Clarivate Analytics; city, Philadelphia; country, USA to eliminate all duplicates. Tiles/abstracts of potential studies will be scanned to exclude all irrelevant literatures. The remaining articles will be identified against all inclusion criteria. Any disagreements between 2 authors will be resolved by a third author through discussion. The process of study selection will be summarized in a flow diagram.

#### 2.5.2 Data collection and management

After study selection, 2 authors will independently collect information from all eligible studies. Any differences will be solved by a third author with consultation. We will extract following information: first author, year of publication, country, sample size, age, sex, CC severity, index test, reference test, outcomes, results, conclusions, and conflict of interest.

#### 2.5.3 Dealing with missing data

Any unclear or missing data will be tried to obtain from original study authors. If we can not request it, we will analyze reachable data using intention-to-treat analysis.

### 2.6 Methodological quality assessment

Two authors will independently appraise methodological quality using Quality Assessment of Diagnostic Accuracy Studies tool.^[20]^ We will assess it through 4 aspects. Any different conflicts will be settled by a third experienced author through consultation.

### 2.7 Statistical analysis

#### 2.7.1 Data synthesis

RevMan V.5.3 software: organization, Cochrane Community; city, London; country, UK and Stata V.12.0 software: organization, StataCorp; city, College Station; country, USA will be employed to carry out statistical analysis. We will estimate outcome values as descriptive statistics and 95% confidence intervals. *I*^2^ statistic will be utilized to check heterogeneity across eligible studies. *I*^2^ ≤ 50% implies homogeneity, and we will use a fixed-effects model. On the other hand, *I*^2^ > 50% reveals discrete heterogeneity, and we will exert a random-effects model. We will estimate values of outcome data using 2 × 2 tables. Additionally, we will estimate a descriptive forest plot and a summary receiver operating characteristic plot. If there is homogeneity among included studies, we will perform a meta-analysis. If there is obvious heterogeneity, we will scrutinize its sources using a subgroup analysis and bivariate random-effects regression approach.

#### 2.7.2 Subgroup analysis

Whenever necessary, we will investigate sources of apparent heterogeneity based on the differences in study characteristics, study quality, and outcomes.

#### 2.7.3 Sensitivity analysis

Whenever necessary, we will examine the stability of study results by eliminating low quality studies.

#### 2.7.4 Reporting bias

If possible, we will perform a funnel plot and Egger regression test to investigate the reporting bias when >10 studies are included.^[21]^

### 2.8 Ethics and dissemination

This study will not collect individual patient data, thus, we will not need ethic approval. We will publish this study on a peer-reviewed journal.

## 3 Discussion

Several studies have reported that Ki67 expression may help to diagnose CC. However, no consistent conclusions have been reached and no study has systematically investigated the diagnostic accuracy of Ki67 expression in patients with CC. Thus, this study will firstly explore the diagnostic accuracy of Ki67 expression in CC. The findings of this study may provide evidence to determine whether Ki67 expression is helpful in diagnose CC.

## Author contributions

**Conceptualization:** Jing Li, Zhi-ye Liu, Hai-bo Yu, Qing Xue, Wen-jie He, Hai-tao Yu.

**Data curation:** Jing Li, Zhi-ye Liu, Wen-jie He, Hai-tao Yu.

**Formal analysis:** Jing Li, Hai-bo Yu, Qing Xue, Wen-jie He.

**Funding acquisition:** Hai-tao Yu.

**Investigation:** Zhi-ye Liu.

**Methodology:** Jing Li, Zhi-ye Liu, Hai-bo Yu, Qing Xue, Wen-jie He.

**Project administration:** Hai-tao Yu.

**Resources:** Jing Li, Zhi-ye Liu, Hai-bo Yu, Qing Xue, Wen-jie He.

**Software:** Jing Li, Zhi-ye Liu, Hai-bo Yu, Qing Xue, Wen-jie He.

**Supervision:** Hai-tao Yu.

**Validation:** Zhi-ye Liu, Hai-bo Yu, Qing Xue.

**Visualization:** Jing Li, Hai-bo Yu, Wen-jie He, Hai-tao Yu.

**Writing – original draft:** Jing Li, Zhi-ye Liu, Hai-bo Yu, Qing Xue, Wen-jie He, Hai-tao Yu.

**Writing – review & editing:** Jing Li, Zhi-ye Liu, Hai-tao Yu.
